# Interacting locally, acting globally: trust and proximity in social networks for the development of energy communities

**DOI:** 10.1038/s41598-023-43608-7

**Published:** 2023-10-03

**Authors:** Rocco Caferra, Annarita Colasante, Idiano D’Adamo, Andrea Morone, Piergiuseppe Morone

**Affiliations:** 1 Department of Economics, Management and Business Law, University of Bari, Bari, Italy; 2 Department of Law and Economics, Unitelma Sapienza University of Rome, Rome, Italy; 3 Department of Computer, Control and Management Engineering, Sapienza University of Rome, Rome, Italy

**Keywords:** Environmental social sciences, Environmental sciences

## Abstract

In this article, we analyze the role of social capital in the formation of sustainable energy communities. Specifically, we study the impact of different dimensions of social capital (i.e., structural, relational, cognitive) in determining willingness to participate in an energy community. Our survey data suggest that social contexts contribute to the development of energy communities, via (at least) two channels: (i) a family path, with individual perspectives showing a partial correlation with those of at least one relative, and (ii) a social channel, with higher social trust and greater interaction with neighbors favoring the propensity to participate in an energy community. The social coordination required for the formation of sustainable energy communities is determined by the quality of social interactions, and the spread of virtuous behavior is determined by not only economic policies (i.e., incentives), but also forward-looking policies favoring local aggregation and the creation of high-quality social capital. Thus, local actions and interactions can contribute to solving global climate change challenges.

## Introduction

Cooperation among active citizens is crucial for the development of effective energy policies^1,2^. As stated by EU directives (i.e., the 2019 Clean Energy Package), energy communities and joint self-consumption programs can increase the number of citizens favoring sustainability on individual and collective, as well as economic, environmental, and societal levels^3^. **Energy communities can support the achievement of sustainable development goals (SDGs)**^4,5^. The literature reports some of the main constraints on energy communities, such as poor institutional support^6^, and deficits in technological systems, including information and communications technology (ICT) infrastructure^7^. Additionally, social and structural arrangements have been recognized as significant for determining the stability of energy communities^3^. Here, stability is understood in economic terms, referring to an appropriate distribution of the gains derived from renewable energy generation. However, changes in socio-technical regimes^8^ from centralized to decentralized energy systems (i.e., with the latter implying local electrical production and consumption) are reliant on not only technical and economic process, but also individual willingness to participate. As behavioral research has widely demonstrated, individuals act “not just for the money”^9^. In fact, personal values and attitudes also contribute to determining pro-environmental engagement^10,11^. Thus, even the most profitable economic policy is likely to be ineffective **under** disinterest and negligent behavior. Besides individual preferences, the social context influences human decisions to cooperate. The so-called peer effect^12^ has been identified as one of the main driver of daily decisions, from political science, psychology, and urban design. As stated in literature^13^, “people who talk together vote together”, and overall people tend to vote the general trend of their neighborhood^14^. Therefore, peer pressure can be considered a driving force in influencing individual decisions, and the willingness to participate into an energy community, being a decision requiring a high level of coordination and cooperation with neighbors, can be determined by both individual and social preferences. This recalls the influences that social norms might have in individual pro-environmental choices, meant as the cardinal “code of conduct” of a group, influencing expectations, opinions, and actions of group members^15^. Existing literature reports interesting evidence about different eco-friendly behavior as consumer choices^16,17^, recycling^18^, and individual energy conservation habit^11,19,20^ while research regarding the impact on the formation of energy communities is still under-explored.

The present study aimed at covering this gap in the literature, considering both the **quality of the social context** and demand factors that drive individual willingness to join an energy community. To that end, we considered a sample of Italian citizens as a case study. In Italy, energy community initiatives are gaining momentum, fostered by monetary incentives. While the stability of energy communities may be limited by poor organization or a lack of economic incentives, shared values and high-quality relationships may contribute to their long-term sustainability. Therefore, in the present study, we applied social capital theories^21^, considering the nature, quality, and quantity of human interactions as potential driving factors for citizen engagement. **Therefore, we used the three-part framework of social capital described by**^21^, comprised of: (i) structural social capital, describing the network ties (i.e. the set of human relationships) that determine the spread of values and opinions; (ii) relational capital, reflecting the quality of social links, in terms of trust; and (iii) cognitive social capital, referring to the set of norms and shared values within the network. We theorized that the resulting set of civic norms, trust, and social relationships could serve as a main driver of social and economic growth^22^, promoting virtuous human paths^23,24^ based on a stable and mutual sharing of environmental values and actions. Sustainable education and trust among the younger generations may foster the development of innovative models for balancing ecosystems, combining socio-economic development with environmental protection^25^ . Energy communities place human activity at the center of all electricity consumption^26^ and, in this way, represent new social models for the ecological transition^27^ . While economic analyses have been applied to quantify the profits associated with these communities, new models are needed to propose the distribution of that wealth. However, such models require a new social paradigm. Accordingly, the present study aimed at assessing the impact of different dimensions of social capital (i.e., structural, relational, cognitive) in determining willingness to participate in an energy community. To this end, we deployed a survey to reconstruct the function of social capital in the formation of energy communities. The remainder of the **article** is organized as follows: Section "Methods" describes the data and methodology, Section "Results and discussion" reports the main results, and Section "Conclusions and policy implications" provides concluding remarks.

## Methods

Data were collected via an online survey that was disseminated using the Prolific platform (www.Prolific.co) between March and April 2023, in a sample of Italians. The focus on Italy was motivated by ongoing policy efforts (i.e., tax incentives) to foster the diffusion of energy communities in that country, despite administrative delays in delivering on the projects. This approach is justified by the literature^28^. A total of 302 observations were randomly collected. All participants who submitted valid answers were rewarded with a small, fixed amount of money. The 36 survey items explored 17 variables. A first draft of the survey was based on the literature on both social analysis^29–31^ and energy communities. The draft survey was then validated by a group of academics and practitioners in the field^25,32^, who provided useful suggestions to improve the structure. The survey was then revised into a final version, based on this feedback. Before answering, people were informed about the broader meaning of energy communities:”An Energy Community is a collection of people who share renewable and clean energy in a peer-to-peer exchange. Energy Communities thus represent an innovative model for the production, distribution, and consumption of energy from renewable sources. This model bases its values on combating energy waste and sharing a basic good at a competitive price, thanks to innovation that is revolutionizing the energy market. You can find more information at: https://www.enelx.com/it/it/storie/2020/05/comunita-energetiche-cosa-sono”. The link was not chosen following commercial policies, but one the most relevant link in google searches in the period it was conducted was chosen, thus following the relevance criteria defined by the Google PageRank algorithm.

Survey questions referred to the following:

- **Response variable** Willingness to join energy communities (WEC). “To what extent do you want to participate into an energy communities?” (10-point value scale)
- **Individual attitude** Individual environmental Concerns. “To what extent do you fell responsible of the climate change?” (10-point value scale)
- **Social Capital**
  - *Structural* Interactions with Friends, relatives and neighbors. “How often do you meet your [one among Friends/ relatives / neighbors]?” (1=”Never”, 2=”once per month”,3=”Less than once per month”, 4=“more than one per month”, 5=“once per week”, 6=“more than once per week”,7=“everyday”)
  - *Relational* Social and Institutional Trust. “Most people can be trusted or never be too careful?” (from 0 to 10). “How much do you trust your nation’s government?” (from 0 to 10)
  - *Cognitive/Civic norms* Friends’ relatives’ and neighbors’ environmental concerns. “To what extent do you think most of your [one among friends/relatives/neighbors] deal with climate change issues?”
- **Control variables** Sex, Age, type of house, description of the surrounding area, marital status, income.

Table 1 presents the descriptive statistics for the abovementioned variables. The sample was young (i.e., average age 31 years) and relatively gender balanced (i.e., 59% male). Approximately 60% of respondents had at least a bachelor’s degree. Nearly 44% of the sample lived in a small city and approximately two-thirds lived in an apartment. The sample presents some deviations from the targeted population (for instance in the gender composition) typical on online survey where the representativeness is difficult to be achieved^33^, that is why the sample selection of Prolific platform has been used to reduce this issue. Social capital was investigated with respect to three dimensions (i.e., structural, relational, cognitive)^21^. While some research has relied on a two-part distinction between what people feel (i.e., cognitive capital) and how they interact (i.e., structural capital)^34^, we further distinguished between the cognitive sphere, referring to a set of shared beliefs and goals within the community (e.g., climate change mitigation involvement), and the relational sphere, referring to trust in other community members. Hence, we considered cognitive capital the set of (pro-environmental) civic norms within the social surroundings.

**Table 1:** Descriptive Statistics.

Variable	Obs	Mean	Std. dev.	Min	Max
Willingness to join energy communities (WEC)	302	6.359	2.352	0	10
Environmental concerns (EC)	302	8.104	1.678	0	10
Structural capital					
Relatives’ interactions	302	4.253	1.82	1	7
Friends’ Interactions	302	4.515	1.518	1	7
Neighborhood’ Interactions	302	3.249	1.91	1	7
Relational capital					
Social trust	302	4.785	2.275	0	10
Institutional trust	302	3.273	2.451	0	9
Civic norms					
Friends’ EC	302	5.556	2.285	0	10
Relatives’ EC	302	4.168	2.556	0	10
Neighbors EC	302	3.818	2.236	0	9
Age	302	31.956	9.846	20	62
Income					
1: 0-15.000 euro	302	0.185	0.389	0	1
2: 15000–30000 euro	302	0.343	0.476	0	1
3: 30000–45000 euro	302	0.219	0.414	0	1
4: 45000–60000 euro	302	0.111	0.315	0	1
5: 60000–75000 euro	302	0.037	0.189	0	1
6: 75000–100000 euro	302	0.03	0.172	0	1
7: more than 100000 euro	302	0.003	0.058	0	1
Prefer to do not disclose	302	0.071	0.257	0	1
House description					
Detached house	302	0.29	0.454	0	1
Block of flats	302	0.663	0.473	0	1
“Villa”	302	0.047	0.212	0	1
Area description					
Small city	302	0.441	0.497	0	1
Countryside	302	0.02	0.141	0	1
City	302	0.229	0.421	0	1
Town	302	0.199	0.4	0	1
Outskirts	302	0.111	0.315	0	1
Marital status					
Unmarried	302	0.788	0.41	0	1
Divorced	302	0.01	0.1	0	1
Prefer to do not disclose	302	0.037	0.189	0	1
Separated	302	0.01	0.1	0	1
Married	302	0.152	0.359	0	1
Widower	302	0.003	0.058	0	1
Education					
Secondary school	302	0.007	0.082	0	1
High school	302	0.414	0.493	0	1
Three-year degree	302	0.266	0.443	0	1
Master’s degree	302	0.286	0.453	0	1
PhD	302	0.027	0.162	0	1
Sex					
Male	302	0.5925	0.492	0	1
Female	302	.397	.490	0	1
Prefer to do not disclose	302	0.01	0.10	0	1

Questions on social capital were adapted from the questionnaires of well-known research agencies, including the European Social Survey (ESS) (https://www.europeansocialsurvey.org/). Structural social capital was investigated via three questions with a common framework: “How often do you meet socially with [friends/relatives/colleagues]?” The inclusion of neighbors was deemed crucial for the definition of bonding capital (i.e., the degree of strong and repeated contact) with potential collaborators in the energy community. While previous studies have emphasized the economic stability of civic cooperation, social stability, indicating joint support and stable relationships with neighbors, may also be highly relevant. In this respect, we considered friends and family proxies of strong, close relationships^35^, since relationships with friends and family are typically characterized by frequent interaction and a contagion of opinion^8,36^. Questions on relational social capital were adapted from the ESS. We considered social trust and trust in institutions (i.e., political trust) the main motivating factors for human engagement in virtuous actions with positive externalities for society^11^. Finally, cognitive social capital was explored considering the pro-environmental attitudes of friends, relatives, and neighbors with whom respondents were strongly and closely tied.

**Figure 1:**
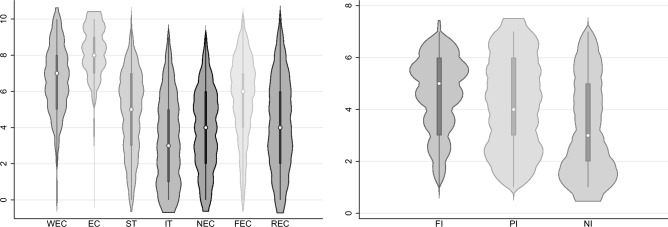
Descriptive violin plot of the main variables of interest. WEC= willingness to join energy communities; EC= Individual Environmental concerns: RI= Interactions with relatives; FI= Interactions with Friends; ST= Social trust; IT= Trust in institutions; FEC= Friends’ Environmental concerns; REC= Relatives’ Environmental concerns; NEC= Neighbors’ Enviornmental Concerns.

Figure 1 presents the distribution of the main variables of interest. WEC was above average, revealing significant individual interest in joining an energy community. Of note, respondents demonstrated a high pro-environmental orientation (EC), slightly above **(t-test** ***p*-value<0.01)** their propensity to join an energy community (WEC). Considering structural social capital, the average value of friend interactions (FI) was higher than that of both relatives (RI) and neighbors (NI) (***p*-value of 0.056 and 0.01 respectively**). With respect to relational capital, social trust (ST) was higher than trust in institutions (IT) (***p*-value<0.01**). Moving to the cognitive sphere, subjects considered their friends more concerned with environmental issues (FEC) than their relatives (REC) and neighbors (NEC) (***p*-value<0.01 in both cases**). On this basis, we tested our research hypothesis, searching for the existence of a positive relationship between the different spheres of social capital and the promotion of energy community initiatives. Considering the nature of the response variable, we opted for an ordered probit model, using an ordinary least squares (OLS) estimate to check robustness (^11,37^). Given the simplicity of exposition and the easy and immediate interpretation of the results, we report and comment on the analyses conducted with the OLS estimator, after checking the consistency of the results with the ordered probit. In fact, the OLS estimator allows us to check the relationship between the independent variables and the dependent without performing further transformations of the coefficients (as is done with the ordered probit). Switching to a linear estimator (OLS) might lead to a loss in accuracy in predicted probabilities (outside the scope of this study), but it can retain the same ability to identify the sign and statistical significance of relationships, as we demonstrate^38,39^.

**Ethics statement** Given that the research is a non-experimental voluntary survey, no ethical approval is necessary^25^. Furthermore, the self-administered survey that is non-experimental in nature was conducted under complete anonymity for the participants, following the legal duty of General Data Protection Regulation (GDPR) (EU) 2016/679. No personal or sensitive information that can be used to identify the respondents were collected. Besides, the consent of the respondents to partake in the online survey were seek before the survey was executed by including an electronic informed consent in the online survey form. All procedures were performed in accordance with relevant guidelines.

## Results and discussion

**In what follows, we present and discuss the main results deriving from the econometric analysis.** In Table 2, the first column (OLS) displays the results of the OLS estimation and the second column (OP) presents the results of the ordered probit. In addition to individual attitudes (which, as expected, positively affected the formation of energy communities), joint energy consumption was also dependent on the social context. Considering the results for the three forms of social capital, at least two transmission channels could be identified: (i) a family path dependence in defining sustainable trajectories (since individual perspectives were partially correlated with those of at least one relative) and (ii) a social channel (since higher social trust and more interactions with neighbors favored the propensity to join an energy community. This suggests that the social coordination required for the formation of sustainable energy communities is dependent on the quality of social interactions. Accordingly, local aggregation and the creation of high-quality social capital might favor long-term and stable cooperation within sustainable communities. Considering the control variables, the surrounding area was relevant to the propensity to join an energy community: respondents who lived in cities (i.e., areas of high population density) were more likely to model low-carbon lifestyles, even though they faced more obstacles^40^. Another interesting result is that the propensity to join an energy community increased with age. Importantly, this finding does not necessarily suggest that younger individuals lack a pro-environmental orientation; rather, it may relate to the precariousness that surrounds their transition to working (and social) life. The creation of an energy community involves significant upfront costs, and this may represent a disincentive to individuals who have not yet decided where they will live over the long term (e.g., many young adults). Further **future research can shed more light on the** trade-off between environmental and social sustainability, as the higher environmental quality that may be achieved via energy communities may come at the expense of higher social inequalities (i.e., by crowding out younger generations). Existing studies report only the benefit in terms of a reduction of energy poverty associated with the development of energy communities^41^ due to a reduction of the energy bill , but the sample socio-demographic characteristics of the citizens involved in this project is also worthy of investigation. On the one hand, the energy cost will be lower for energy community participants, on the other hand this effect might exacerbate economic inequality and disparities with those not taking part into the project. To the best of our knowledge, this is the first study raising this point.

**Table 2:** Regression results. Robust standard errors are in parentheses.

Dependent variable: WEC	OLS	OP
Individual attitude		
Environmental concerns (EC)	.716*** (0.089)	.423*** (0.053)
Structural social capital		
Relatives’ interactions	−0.064 (0.065)	−0.033 (0.036)
Friends’ interactions	−0.08 (0.075)	−.071 (0.063)
Neighbors’ interactions	0.103* (0.059)	0.051* (0.034)
Relational social capital		
Social trust	.177*** (0.069)	.102*** (0.037)
Institutional trust	−0.048 (0.06)	−0.025 (0.035)
Civic norms		
Friends’ EC	−0.036 (0.07)	−0.019 (0.04)
Relatives’ EC	.150*** (0.057)	.094*** (0.034)
Neighbors’ EC	−0.045 (0.066)	−0.022 (0.036)
Control variables		
Log age	1.148** (0.53)	.579* (0.305)
Income		
2	−0.413 (0.35)	−0.203 (0.202)
3	−0.114 (0.347)	−0.073 (0.198)
4	0.503 (0.446)	0.32 (0.268)
5	−0.574 (0.52)	−0.313 (0.309)
6	0.365 (0.646)	0.178 (0.379)
7	0.571 (0.758)	0.395 (0.441)
Prefer to do not disclose	0.025 (0.46)	−0.007 (0.259)
House description		
Block of flats	−0.177 (0.248)	−0.079 (0.141)
Villa	0.727 (0.578)	0.467 (0.327)
Area description		
Countryside	−0.616 (0.626)	−0.417 (0.363)
City	.529* (0.305)	.330* (0.176)
Town	0.155 (0.311)	0.1 (0.177)
Outskirts	0.37 (0.348)	0.199 (0.194)
Marital status		
Not Married	0.182 (0.817)	0.023 (0.396)
Divorced	−2.077* (1.232)	−1.274** (0.669)
Separated	−0.685 (0.952)	−0.608 (0.479)
Married	0.186 (0.902)	0.122 (0.454)
Widowed	−5.632*** (0.985)	−2.571*** (0.552)
Education		
Secondary school	0.079 (0.782)	−0.006 (0.425)
High school	−0.156 (0.499)	−0.078 (0.268)
Three-year degree	0.002 (0.523)	0.032 (0.278)
Master’s degree	−0.034 (0.521)	0.012 (0.276)
Sex		
Male	−0.146 (0.135)	0.935 (0.731)
Prefer to do not disclose	0.935 (0.731)	1.7853 (1.459)
Constant	−3.529 (2.449)	
Observations	302	302
R-Squared	Adj: 0.465	Pseudo: 0.1361

***p< .01, **p< .05, *p<.1.

Achievement of some SDGs can be achieved by pursuing the model of energy communities and optimal use of natural resources. However, if we use green energy, this should not cause people to consume resources when it is not necessary^42^ . The present findings provide new evidence on the social impact of energy communities—an aspect that is under-reported in the literature^43^. Certain regulatory acts may slow the development of collective prosumers^44^, and some organizational models may impose further constraints. However, these aspects mainly apply to energy communities (which require new forms of participatory governance), rather than collective self-consumption programs (in which individuals act as agents of change)^45^. Some analyses have quantified the cost and emission reductions that accompany the development of energy communities^46^, confirming a strong focus on environmental and climate change issues^47^. Nevertheless, the sustainable transition in Italy cannot be driven solely by contributions from small entities. The first energy communities in Italy have produced promising data, and incentives are planned to encourage their development (i.e., 40% grants to municipalities with fewer than 5000 residents). The present findings, however, show a greater potential for energy communities in urban contexts. In this regard, a balance **should** be struck between buildings and open spaces, as this may play a decisive role in the urban energy transition^48^. Additionally, as the conflicting interests of different actors could make energy communities less stable^3^, increased end-consumer participation and shared benefits should be pursued^49^. In this regard, economic models **should** be identified to ensure an appropriate distribution of benefits and to find compromise solutions to satisfy different renewable self-consumers with unique energy habits^50^. The economic advantages of energy communities are strongly impacted by the percentage of self-consumed energy and avoided costs in the bill - aspects that are even more delicate in the current period of sharply rising electricity costs^27^. Economic factors have been shown to have a significant impact on prosumer behavior^29^, and some studies have placed awareness and personal and social norms on equal footing^51^. Thus, once policies are able to control for market risks^52^ and ensure economic benefits for prosumer behavior, they should turn their focus to social aspects. As the Italian energy context is dependent on imports, socio-economic issues related to a loss of fossil fuel production revenue should not have an effect on prosumers^53^. In the present study, participants expressed an interest in joining an energy community. This result may be explained in reference to multiple aspects, beyond climate concerns. Specifically, energy communities are associated with both economic and environmental benefits. Economically, the benefits are highly attractive in light of current energy bills, which have forced the Italian government to intervene with economic support to citizens and firms. **From the environmental perspective**, the benefits align with Italian citizens’ desire for change and sustainability, across all sectors. It is often argued that the younger generations are most attentive to environmental issues. However, in the present study, younger participants did not lead in positive behaviors. It may be the case that young adults are held back from joining energy communities due to the high upfront costs, which may never be recuperated (if, e.g., the individual moves to another location). Young adults value-and often require-flexibility, and this may prevent them from making long-term choices and commitments. Additionally, it is frequently the case that young adults live with family members or rent private accommodation, and this may limit their understanding of the economic costs of energy. A policy proposal to tackle this issue could aim at creating rental housing within energy communities, in university locations. In particular, research has shown that students perceive energy communities as strategically important for decarbonization; further training could reinforce this belief^25^. **Educational aspects are essential to foster the development of a mindful attitude toward sustainable issues**^54,55^. In the literature, energy communities are mainly conceptualized as places, rather than participatory processes. In addition, greater emphasis is placed on their contribution to economic goals, rather than their social or political aims^56^. Training programs should aim at conveying energy communities more broadly and accessibly. While the basic idea of energy communities is that electricity generated from renewable sources (e.g., solar) provides economic benefits and contributes to mitigating climate change, the effectiveness of these communities is reliant on the existence of a strong social framework. Thus, policy choices must prevent a scenario in which low-income individuals cannot install photovoltaic (PV) systems or join energy communities or collective self-consumption schemes, due to affordability concerns. Furthermore, energy communities must be framed within a broad reality that includes storage systems^57^ and circular models to recover end-of-life products for future manufacturing^27^ . **To this end, circular business models associated with blockchain can make an important contribution**^58,59^. The present findings contribute to the literature and identify new insights from a social perspective. The results align with human organization theory, showing that individuals may fulfill personal needs for protecting the environment within an organizational context that is narrow (i.e., including family members), moderate (i.e., including friends), or wide (i.e., including neighbors) in scope. In energy communities, a wide perimeter is crucial. Thus, further research **should** be conducted to support communication models, so that field experiments with shared, achievable goals may be better understood in practice. To this end, new professionals (i.e., young graduates) may be tasked with creating regional social communities and communication channels (not limited to the digital sphere). Additionally, the structural and cognitive aspects that characterize friendships may be extended to other types of relationships, fostering communities of discussion and stakeholder engagement^60^, aimed at identifying solutions. Human activity is at the heart of electricity consumption^26^, and social capital and human resources are at the heart of energy communities^61^. Such communities are strengthened by shared values, which materialize first in the household (see Table 2). In addition, socio-institutional factors are highly relevant, as energy communities^62^ are legal entities within a larger socio-legal institution^63^. The present finding of greater social trust, relative to political trust, is not surprising, considering the low level of public participation in the most recent general elections in Italy. Thus, political engagement **should** be strengthened. Shared participatory models, immediate decision-making, future-oriented choices, and the realization of sustainable communities are all elements on which to build trust in politics. Additionally, certainty about incentive policies, reduced bureaucracy, and fewer constraints towards the power grid would further improve the implementation of autonomous and decentralized electricity systems.

## Conclusions and policy implications

The development of energy communities is crucial for the transition towards sustainable energy systems. Such communities promote economic, environmental, and social goals. However, their success is dependent on not only technical and economic factors, but also individual willingness to participate and the social context. To address these latter factors, it is essential to consider social capital theories and the role of human interaction quality.The present study generated important findings regarding the importance of cooperation among active citizens for the development of energy policies, and the significance of social capital in driving individual willingness to join an energy community. First, we found that social capital theories-considering structural social capital, relational capital, and cognitive social capital^21^-are useful for understanding the nature, quality, and quantity of human interactions, which drive engagement in energy communities. Second, we found that joint energy consumption depends on the social context, and the family and social channels are key for influencing willingness to participate in an energy community. Furthermore, individuals who live in a city environment are more likely to model low-carbon lifestyles, and the propensity to join an energy community increases with age. Young adults may be held back from joining an energy community due to financial constraints and a lack of long-term commitment. To tackle this issue, we propose a policy to develop rental housing within energy communities in university locations. Our study also highlighted the need for a broader framing of energy communities, considering both storage systems^57^ and circular models^27^. Additionally, it emphasized the importance of stakeholder engagement and communication channels, which may be achieved by involving young adults in the creation of regional social communities and fostering communities of discussion. Finally, we found that socio-institutional factors are relevant to energy communities^62^, as such communities are legal entities within a larger socio-legal institution^63^. To build trust in politics, shared participatory models, future-oriented choices, and the realization of sustainable communities are essential. Additionally, certainty about incentive policies, reduced bureaucracy, and fewer constraints towards the power grid are needed for the effective implementation of autonomous and decentralized electricity systems. In conclusion, the development of energy communities requires a multifaceted approach, considering not only technical and economic aspects, but also social, cultural, and organizational factors. By incorporating these elements, energy communities may effectively contribute to the transition towards sustainable energy system and promote a more equitable and environmentally conscious society.

## Data Availability

The dataset generated during and/or analysed during the current study are available from the corresponding author on reasonable request.
